# BRCA testing and management of BRCA-mutated early-stage breast cancer: a comprehensive statement by expert group from GCC region

**DOI:** 10.3389/fonc.2024.1358982

**Published:** 2024-04-25

**Authors:** Humaid O. Al-Shamsi, Ahmed Alwbari, Fathi Azribi, Francois Calaud, Sanjay Thuruthel, Syed Hammad Hassan Tirmazy, Sharif Kullab, Sonia Ostomane, Omalkhair Abulkhair

**Affiliations:** ^1^ Burjeel Medical City, Burjeel Holding, Abu Dhabi, United Arab Emirates; ^2^ Gulf Medical University, Ajman, United Arab Emirates; ^3^ Emirates Oncology Society, Dubai, United Arab Emirates; ^4^ College of Medicine, University of Sharjah, Sharjah, United Arab Emirates; ^5^ Gulf Cancer Society, Alsafa, Kuwait; ^6^ Almoosa Specialist Hospital Cancer Center, Al Ahsa, Saudi Arabia; ^7^ American Hospital, Dubai, United Arab Emirates; ^8^ National Center for Cancer Care & Research, Doha, Qatar; ^9^ Kuwait Cancer Control Centre, Kuwait, Kuwait; ^10^ Consultant Oncologist and Acting Head of Oncology Dubai Hospital, DHA, Dubai, United Arab Emirates; ^11^ King Khalid University Hospital, Riyadh, Saudi Arabia; ^12^ Cleveland Clinic, Abu Dhabi, United Arab Emirates; ^13^ Dr. Sulaiman Al Habib Medical Center, Riyadh, Saudi Arabia

**Keywords:** breast cancer, *BRCA1*, *BRCA2*, GCC, TNBC, olaparib

## Abstract

BReast CAncer (*BRCA*)1 and *BRCA2* gene pathogenic variants account for most hereditary breast cancers (BC). Identification of *BRCA* mutations can significantly influence both prognosis and treatment outcomes. Furthermore, it enables the identification of individuals who are at heightened risk of developing BC due to inherited genetic mutations. Many developing countries rely on western guidelines for *BRCA* testing and BC management; however, there exist wide disparities in the prevalence of risk factors, availability of medical resources, and practice patterns. Guidelines tailored to specific regions can help mitigate healthcare variations, promote consistency in treatment, and aid healthcare providers in identifying effective therapies for improving patient outcomes. Hence, oncologists from the Gulf Cooperation Council (GCC) congregated virtually in March 2023 and reviewed existing data on the epidemiology of BC, *BRCA* mutations, practices and challenges associated with *BRCA* testing and management of *BRCA* mutated early-stage BC in the GCC region. They also provided insights on the real-world diagnostic and treatment practices and challenges in the GCC region in the *BRCA*-mutated early-stage BC domain and suggested some variations to international guidelines to aid their uptake in this region.

## 1 Introduction

Breast cancer (BC) is the most commonly diagnosed cancer and the leading cause of cancer death among women in the Gulf Cooperation Council (GCC) region, with an age-standardized incidence rate of 34.4 per 100 000 and a mortality rate of 10.6 per 100 000 in 2020 (**Supplementary Table S1**) (1). In most GCC countries, the incidence of BC has increased over time among women (2). Hereditary factors are responsible for around 10% to 30% of BC cases (3) and 16% of these hereditary cases are related to germline mutations in BReast CAncer gene (*BRCA*)*1* and *BRCA2* genes (4). Other factors such as early age menarche, later age at menopause, shorter breastfeeding periods, use of oral contraceptives or hormonal therapy, dense breasts, and older age are found to be associated with increased risk of BC (5, 6).

Compared to the Western population, BCs have diverse clinical, pathological and molecular features including early onset, higher tumor grade, higher human epidermal growth factor receptor (HER)2 amplification rate, more aggressive subtypes and a lower rate of luminal subtype, in the GCC population (7–9). Evidence suggests that approximately 46.2% to 54% of BC patients are diagnosed at advanced disease stage (7, 8, 10–12), 23.3% to 28% are diagnosed with localized tumors while ≤2% with *in-situ* carcinoma (8, 10). In the GCC region, the vast majority of BC cases (82.1% to 93%) have invasive ductal carcinoma (IDC) (7, 8), and 19.2% to 29.5% have HER2 overexpression (7, 8, 12), while 14.3% to 26.9% have triple-negative BC (TNBC) (7, 8). The average age of patients at the presentation of BC is at least a decade younger in the GCC population compared to the Western population (<48 years vs 60 years) (7, 13).

Outcomes in BC depend primarily on timely diagnosis and access to appropriate treatment. Patients who are diagnosed at the early stages (stage 0, I, II) tend to have higher overall survival (OS) rates than people diagnosed with stage III or IV BC (14); the 5-year survival rate reported for women with stage I BC was found to be 99% and the same for patients with stage II BC was 86% (14). The 5-year survival rate in the GCC region ranges between 63% and 89%, with the highest 5-year survival rate being reported in the United Arab Emirates (UAE) and the least being reported in Bahrain (15–17). The cumulative risk for developing BC by age 70 years was 65% for *BRCA1* carriers and 45% for *BRCA2* carriers (18). Identification of *BRCA* mutation in a woman diagnosed with BC may have an impact on both prognosis and treatment (19)—especially it influences the extent of surgery such as the choice of breast-conserving surgery (BCS) or contralateral mastectomy, also predicts the effectiveness of platinum-based chemotherapy (20) and poly (ADP-ribose) polymerase (PARP) inhibitors (21). Moreover, it facilitates the identification of individuals who are at high risk of BC due to hereditary genetic mutations. This knowledge can help with making decisions regarding risk-reducing measures such as enhanced surveillance, prophylactic surgery, and chemoprevention. Although most developing countries lean on western guidelines for the management of BC, there are wide differences in the prevalence of risk factors, availability of medical resources, and practice patterns. Region-specific guidelines can curb healthcare variations, drive consistency in delivery, and help healthcare providers navigate effective therapies for improving patient outcomes. Hence, this expert opinion paper intends to provide data on the epidemiology of BC, *BRCA* mutations, practices, and challenges associated with *BRCA* testing in the GCC region. It will also provide recommendations for the *BRCA* testing and management of *BRCA*-mutated early-stage BC.

## 2 Methodology

A multidisciplinary panel of 9 oncologists, experts in BC, from 4 different GCC countries (Kuwait [n=1], Kingdom Saudi Arabia (KSA) [n=3], Qatar [n=1], and UAE [n=4]) congregated virtually in March 2023 to discuss gaps observed in the clinical practice and treatment goals in patients with *BRCA* mutated early-stage BC in GCC region. The aim was to gain insights into the evolving treatment paradigm in germline *BRCA*-mutated early-stage BC. The panel discussed the available data on disease burden, *BRCA* mutations (*BRCA*m), *BRCA* testing, and management practices along with associated challenges specific to their region. They provided strategic as well as implementable recommendations to enhance *BRCA* testing in early-stage BC in the GCC region. Additionally, members of the panel also provided recommendations for developing a treatment algorithm for *BRCA*-mutated early-stage BC. We present an expert opinion manuscript with recommendations for the *BRCA* testing and management of *BRCA* mutated early-stage BC in GCC, based on the published literature and expert clinical opinion. All the experts critically reviewed, revised, and approved the manuscript draft.

## 3 Germline *BRCA1* or *BRCA2* mutations and their implication on the prognosis and management of BC

Germline mutations in *BRCA1/2* are found in 3% to 4% of all women with BC, including 10% to 20% of those with TNBC (22). The cumulative risk of developing BC by age 80 years is 72% (95% confidence interval [CI], 65% to 79%) and 69% (95% CI, 61% to 77%) in those harboring *BRCA1* and *BRCA2* mutation (23), respectively compared to 13% risk in the general population (24).

Patients with *BRCA-*mutated BC have distinct tumor characteristics, often characterized by a higher tumor-grade (25) with special immunophenotypic features (25–27), poorly differentiated infiltrating ductal carcinomas and a more aggressive phenotype (often triple negative/invasive ductal carcinomas) (25, 26), compared with the sporadic population (28, 29). In addition, patients who harbor *BRCA1/2* mutations are more frequently diagnosed with BC at an early age (*BRCA1* at 35 years and *BRCA2* at 40 years) compared with those with sporadic disease (54 years) (25).

A meta-analysis demonstrated a significantly higher risk for ipsilateral breast recurrence (IBR) in *BCRA1/2* mutation carriers compared to non-carriers following BCS at a median follow-up ≥7 years (30). Contralateral BC is more often observed in *BRCA*-mutated BC than in sporadic BC (31). Several studies demonstrated that *BRCA*-mutated BC has worse OS (32) and BC-specific survival (BCSS) than sporadic/*BRCA*-negative cases (33).

Emerging research on BC demonstrates that *BRCA* status predicts sensitivity to platinum-based chemotherapy (20), and PARP inhibitors (21), owing to the ability of these drugs to inhibit deoxyribonucleic acid (DNA) repair pathways. Evaluation of *BRCA1/2* mutational status (33) in patients with BC helps to potentially expand treatment options, implement prevention strategies, and improve survival outcomes (34).

## 4 Epidemiology and prevalence of *BRCA* mutations in GCC

Except for Kuwait, the prevalence of a germline *BRCA*m in GCC countries ranges from 10% to 12% in unselected BC patient populations (35–38). In Kuwait germline *BRCA1* mutation prevalence rate was reported as 21% (39). However, this finding in Kuwait could be due to a small sample size and selection bias; it should not be considered robust enough to affect the clinical practice. *BRCA* mutation prevalence is higher in patients with a family history of BC diagnosed at a young age or a family history of ovarian cancer (40).

## 5 Management of *BRCA*-mutated early-stage breast cancer

### 5.1 Role of MDT and genetic counselor

#### 5.1.1 Role of MDT

Multidisciplinary teams (MDT) play a critical role in the early management of BC. The MDT approach is recommended by many international guidelines (41, 42).

MDT approach presents a significant impact on patient management (43). Patients discussed at MDT meetings are more likely to receive more accurate as well as complete pre-operative staging, and neo-adjuvant/adjuvant treatment (44). MDT care can intercept 98.8% of all medication errors, thereby improving the quality of care (45). In women with early BC, it has the potential to improve quality of life, reduce mortality, and reduce healthcare costs (46). Studies have reported that patients who are managed by MDTs have improved survival outcomes (47) and the relative risk of recurrence (hazard ratio: 0.84; 95% CI, 0.70 to 0.99) and death (hazard ratio: 0.89; 95% CI, 0.82 to 0.96) was significantly decreased compared to those who are not (48).

#### 5.1.2 Role of genetic counselor

A genetic counselor plays a crucial role in early BC management. Genetic counseling before genetic testing is endorsed by many international guidelines (41, 42). Genetic counseling has been shown to improve patient outcomes with positive downstream effects as patients are more equipped to share the results of genetic tests with extended families (49). Some key aspects of their role in BC management are illustrated in **Supplementary Figure S1**.

### 5.2 Diagnostic work up

Breast cancer is commonly diagnosed either through screening or a symptom (e.g., pain or a palpable mass) that prompts a diagnostic examination. Mammography (bilateral) is the standard diagnostic modality for diagnosing BC (41). However, false-negative mammography results are often observed in some cases. Studies reported that false-negative mammography results are associated with factors such as higher breast tissue density, the presence of *BRCA1/2* mutations and, both of which may be more prevalent in younger women (50). In such cases (high-risk patients), augmenting mammography with ultrasound can uncover additional cases of mammographically hidden cancers; the use of magnetic resonance imaging (MRI) is optional. The sensitivity of ultrasound screening appeared to be similar to that of mammography in a population with a high-risk of BC (41, 51, 52). Breast MRI may be used for staging evaluation to define the extent of cancer, in the adjuvant or neo-adjuvant settings to detect the presence of multifocal or multi-centric cancer in the ipsilateral breast, or as screening of the contralateral BC at the time of initial diagnosis (41).

Routine pathologic evaluation of the primary tumor and cytology/histology of the axillary nodes, if involvement is suspected remains the most critical element in determining the prognosis of patients with BC (53). Pathological diagnosis should be based on a core needle biopsy, preferably obtained by ultrasound or stereotactic guidance (53). Additionally, the analysis of specific biomarkers, such as hormone receptors (estrogen and progesterone receptors) and HER2 are important in guiding targeted therapies (53). HER2 testing is routinely recommended in all cases of invasive BCs (41).

#### 5.2.1 Genetic testing for *BRCA* mutations

The National Comprehensive Cancer Network^®^ (NCCN^®^) (54), and several other professional organizations (53, 55) recommend genetic testing for patients who are at high risk for harboring a pathogenic mutation in one of the BC–predisposition genes. These organizations have developed criteria based on personal/family history and age of onset of cancer to identify patients at high risk (**Table 1**) (53–56, 62). The majority of these guidelines are primarily based on the probability of carrying pathogenic mutations in *BRCA* genes. Some of these guidelines (55) propose the use of screening tools (57–61) to identify a family history associated with an increased risk for potentially harmful mutations in BC-susceptibility genes (*BRCA1* or *BRCA2*). Recent studies indicated that nearly 50% of women with BC with germline predisposing mutations are missed by current testing criteria (63). Furthermore, family history–based criteria have limited pertinency in patients who are adopted or unaware of the family history of cancer or have limited family structure (64). Studies on universal testing indicate that guidelines should be broadened to encompass testing of all patients diagnosed with BC (65–67). Researchers report that universal genetic testing after BC diagnosis can uncover clinically significant germline pathogenic variants that might otherwise escape detection due to narrow selection criteria as per current testing guidelines (66).

**Table 1:** International guidelines recommendations for genetic or *BRCA1/*2 testing.

Guidelines	Recommendations for testing high-penetrance breast cancer susceptibility genes (including *BRCA1/2*)
NCCN, 2024 (54)	Individual with •→ Known pathogenic or likely pathogenic variant in the family •→ Meeting the criteria below but who tested negative with previous limited testing (eg, single gene and/or absent deletion duplication analysis) and are interested in pursuing multi-gene testing •→ Known pathogenic or likely pathogenic variant on tumor genomic testing that has clinical implications if also identified in the germline •→ Who meets Li-Fraumeni syndrome (LFS) testing criteria or Cowden syndrome/PTEN hamartoma tumor syndrome testing criteria •→ Personal history of BC o→ ≤50 years o→ Any age: ▪→ Treatment indications – To aid in systemic treatment decisions using PARP inhibitors for BC in the metastatic setting – To aid in adjuvant treatment decisions with olaparib for high-risk, HER2-negative BC ▪→ Pathology/histology – TNBC – Multiple primary BCs (synchronous or metachronous) – Lobular BC with a personal or family history of diffuse gastric cancer ▪→ Male BC ▪→ Ashkenazi Jewish ancestry ▪→ Family history – ≥1 close blood relative with ANY: o→ BC at age ≤50 years o→ male BC o→ ovarian cancer o→ pancreatic cancer o→ prostate cancer with metastatic, or high- or very-high-risk group – ≥3 total diagnoses of BC in patient and/or close blood relatives – Individuals affected with breast cancer (not meeting testing criteria listed above) or individual unaffected with breast cancer with a first- or second-degree blood relative meeting any of the criteria listed above
ESMO, 2019 (53)	•→ Strong family history of breast, ovarian, pancreatic and/or high-grade/metastatic prostate cancer •→ Diagnosis of BC before the age of 50 years •→ Diagnosis of TNBC before the age of 60 years •→ Personal history of ovarian cancer or second BC or male sex
NICE, 2019 (56)	*Referral to a Specialist Genetic Clinic* •→ An individual with the following family history of female BC: o→ Two first-degree or second-degree relatives diagnosed with BC at a younger age <50 years (at least one must be a first-degree relative) o→ Three first- or second-degree relatives diagnosed with BC at younger age <60 years (at least one must be a first- degree relative) o→ Four relatives diagnosed with BC at any age (at least one must be a first-degree relative) •→ Families containing one relative with ovarian cancer at any age and on the same side of the family: o→ One first-degree relative (including the relative with ovarian cancer) or second-degree relative diagnosed with BC at younger than age 50 years o→ Two first-degree or second-degree relatives diagnosed with BC at younger than the average age of 60 years o→ Another ovarian cancer at any age •→ Families affected by bilateral cancer (each BC has the same count value as one relative): o→ One first-degree relative with cancer diagnosed in both breasts at a younger age (<50 years) o→ One first-degree or second-degree relative was diagnosed with bilateral cancer and one first- or second- degree relative or diagnosed with BC at a younger age (<60 years) •→ Families containing male BC at any age and, on the same side of the family, at least: o→ One first-degree or second-degree relative diagnosed with BC at a younger age <50 years o→ Two first-degree or second-degree relatives diagnosed with BC at a younger age <60 years •→ A formal risk assessment has given risk estimates of: o→ 10% or greater chance of a gene mutation being harbored in the family o→ Greater than 8% risk for developing BC in the next 10 years o→ 30% or greater lifetime risk for developing BC
USPSTF, 2019 (55)	•→ Women with a family history of breast, ovarian, tubal, or peritoneal cancer. Screening can be done using one of several screening tools designed to identify a family history that may be associated with an increased risk for potentially harmful mutations in BC-susceptibility genes *(BRCA1* or *BRCA2*). •→ Women with positive screening results should receive genetic counseling and, if indicated *BRCA* testing. •→ Tools evaluated by the USPSTF include the Ontario Family History Assessment Tool (57), Manchester Scoring System (58), Referral Screening Tool (59), Pedigree Assessment Tool (60), and Seven-Question Family History Screening (61).^,*^
ASBrS, 2019 (62)	•→ Genetic testing should be available to all patients with a personal history of BC. •→ Patients who had genetic testing previously may benefit from updated testing for PALB2, genomic rearrangements in *BRCA1/2*, and other potentially relevant genes, if not performed already. •→ Genetic testing should be made available to patients without a history of BC who meet NCCN Clinical Practice Guidelines in Oncology (NCCN Guidelines^®^).

ASBrS, American Society of Breast Surgeons; BC, breast cancer; NCCN=National Comprehensive Cancer Network^®^ (NCCN^®^); NICE, National Institute of Clinical Excellence; TNBC, triple-negative breast cancer; USPSTF, U.S. Preventive Service Task Force.

*Each risk assessment tool has its own strengths and weaknesses; clinicians should be aware of these before use.

†Cancer of the peritoneum and fallopian tubes should be considered a part of the spectrum of hereditary breast and ovarian cancer syndrome.

††Close relative is defined as a first−degree relative (mother, sister, daughter) or second−degree relative (grandmother, granddaughter, aunt, niece).

*BRCA1* and *BRCA2* gene mutations account for most actionable genetic BC predispositions and are increasingly used for personalized BC management and PARP inhibitors therapy of *BRCA*-related cancer (68). *BRCA1* and *BRCA2* mutations are found to be associated with a younger age of onset (25). Also, in GCC countries, the mean age at diagnosis was less than 48 years (with 69% of cases between 25-54 years) (7, 13). Thus, the experts have proposed the criteria mentioned in **Box 1** for *BRCA* testing.

Research investigating next-generation sequencing workflows for *BRCA1/2* genes in samples associated with hereditary breast and ovarian cancer has shown outstanding performance, achieving nearly 100% sensitivity and specificity, while also proving to be cost-effective when compared to single-site mutation testing specifically for these genes (69).

Diagnostic laboratories have identified numerous variants of uncertain (or unknown) significance (VUS) in the *BRCA1* and *BRCA2* genes, owing to their large size and the extensive screening conducted on them. One study reported a VUS frequency rate for *BRCA1* and *BRCA2* of 13% for 10,000 consecutive individuals (70). Studies from the GCC region reported a high rate of VUS ranging from 14.5% to 25.4% (36, 38). However, initiatives to reclassify *BRCA* VUS are likely to reduce this number (71). Evaluating a VUS in *BRCA* genes is a complex undertaking, but it is reported that VUS can be characterized by gathering evidence from databases that document well-characterized populations, and in silico assessment (71).

#### 5.2.2 Impact of timing of genetic testing on surgical decision

Traditionally, *BRCA* testing is conducted after primary surgery for BC; later once testing results are available, patients identified with *BRCA* mutations undergo a second breast surgery/bilateral salpingo-oophorectomy for risk reduction. Recent advancements in genetic testing have significantly reduced turnaround time for *BRCA1/2* mutation tests. This has allowed patients to undergo genetic testing without the need to delay treatment. Consequently, integration of test results into management decisions can now be seamlessly achieved at the time of diagnosis. This may eliminate the requirement for a second breast surgery for risk reduction, as some women diagnosed with deleterious mutations choose to concurrently undergo therapeutic surgery for the affected breast and risk-reducing surgery for the contralateral breast.

Studies indicated that genetic diagnosis before surgery has an impact on the surgical decision, choosing unilateral mastectomy or bilateral mastectomy in *BRCA* mutation carriers with BC (72). A study conducted on patients with unilateral BC reported that only 14.7% of patients with unknown *BRCA* mutation status before surgery received contralateral mastectomy in contrast to 76.4% of patients who underwent contralateral prophylactic mastectomy with *BRCA* mutation status known preoperatively. These data support preoperative genetic testing for *BRCA* mutation in patients with newly diagnosed BC to enable appropriate planning of surgical treatment decisions (73). Hence, providing genetic counseling and *BRCA* testing before surgical approach and developing treatment strategies for patients with a high risk of BC is important (72).

#### 5.2.3 Current *BRCA* testing landscape, challenges, and expert recommendations for improving

In the GCC region, there are variations in practices for *BRCA* testing. Currently, few accredited loco-regional laboratories perform *BRCA* testing. In most countries limited number of cancer centers have a dedicated genetic counselor; counseling is often provided by the medical oncologist, with evident variations in skills and knowledge in this niche area of expertise. Financial support for *BRCA* testing is provided by pharmaceutical companies. In this region, very few patients are referred for *BRCA* testing, particularly in government hospitals because most patients neither have insurance nor are willing to pay for the test and the referral is left at the discretion of individual physicians, surgeons, oncologists, and finally patients, which causes inconsistency of *BRCA* testing. This has resulted in the lack of a regional integrated genetic database. In government hospitals, there are constraints concerning indications, the budget, and a long waiting list for performing screening tests. However, in private institutions, *BRCA* testing is being endorsed for almost all or high-risk BC patients who have insurance or are willing to pay for the tests. In some countries, genetic testing is often excluded from health insurance policies, making it difficult for patients with cancer to access this crucial service. At present, there are no region-specific genetic testing algorithms or guidelines that regulate *BRCA* testing in the GCC region. Currently, NCCN Guidelines^®^ (41) are followed for recommending germline *BRCA* testing. Universal genome testing (wherever possible) or *BRCA* testing for an extended population with indications beyond those in the guidelines is advised by the majority of experts considering the treatment-related benefits of *BRCA* testing and it is implemented in some centers in the GCC, yet we believe there are few centers offering universal testing.

A correspondence article published in 2016 reported that in GCC, most molecular diagnostic samples were sent to Western countries for testing and analysis, and the results from a significant amount of these samples came back negative or inconclusive (74). This finding warrants an immediate need to establish accredited molecular diagnostics locally to customize the molecular genetic approaches. Real-world data have suggested a significant deficit in physician-driven referrals for *BRCA* testing in guideline-eligible BC patients primarily due to a lack of access and knowledge about the criteria for testing (75). Less than 60% of guideline-eligible patients with BC received *BRCA* testing in the United States of America (USA) and European countries (75, 76). In contrast, 97% of guideline-eligible BC patients received *BRCA* testing in Israel (76). Lack of knowledge among community oncologists/surgeons about the selection criteria is one of the important barriers for implementing *BRCA* testing in the GCC region. Expert recommendations for *BRCA* testing and for supportive measures for improving *BRCA* testing in the GCC region are present in **Box 1.1** and **Box 1.2**.

Box 1.1 **Expert recommendations for *BRCA* testing in the GCC region.**

- Genetic counseling and testing for germline *BRCA1* and *BRCA2* mutations should be indicated in the following scenarios:

Individual (BC)

- Age less than or equal to 50 years

- Triple-negative BC at any age

- Bilateral BC at any age

- HR-positive BC with N2 disease at any age

- BC with Ashkenazi Jewish or Icelandic heritages

- Any patient who is eligible for adjuvant PARP inhibitor

- Male BC

Positive family history is defined as below:

- ≥1 close blood relatives with BC at age ≤ 50

- ≥1 close blood relative with male BC

- ≥1 case of a blood relative with ovarian cancer

- ≥1 case of a blood relative with pancreatic cancer

- ≥1 case of a blood relative with prostate cancer with metastatic, or high- or very-high-risk group

- ≥3 diagnoses of breast or prostate cancer (any grade) on the same side of the family including the patient with BC

- Individuals affected with BC (not meeting testing criteria listed above) or individual unaffected with BC with a first- or second-degree blood relative meeting any of the  criteria listed above

BC, Breast cancer; *BRCA*, BReast CAncer gene; HR, Hormone receptor; PARP, poly-ADP ribose polymerase

Box 1.2 **Expert recommendations for supportive measures for improving *BRCA* testing in the GCC region.**

  ▪ Need refined definitive region-specific guidelines for *BRCA* testing

▪ Raise awareness among community oncologists/surgeons about the selection criteria for *BRCA* testing

▪ Increased access to testing would likely lead to more patients pursuing testing and improving rates of identification of gene carriers

▪ All institutions should have access to genetic counselors who are experienced in counseling patients with BC

▪ Regulators and stakeholders should work to make *BRCA* testing widely available and accessible for BC patients

▪ Systematically discuss the cases of variants of unknown significant mutations with a genetic counselor in an MDT approach

▪ For patients with VUS, genetic counselors need to follow up with the patients to check if they develop any other cancer in their family

▪ Create a database for *BRCA* variants and *BRCA* pathogenic variants and compare it with the global data

▪ There is an urgent need for extensive, well-controlled, genetic epidemiological studies to provide accurate *BRCA1* and *BRCA2* mutation prevalence among patients with BC in the GCC region

▪ Establish a centralized laboratory that provides free *BRCA* tests and delivers timely test results without discrepancies

▪ It is recommended to make *BRCA* testing available for all patients whenever possible. An approach to generalize *BRCA* testing to the general population with BC instead of restricting it to only a group of patients with BC (per the selective criteria) to provide more treatment benefits.

BC, Breast cancer; *BRCA*, BReast CAncer gene; GCC, Gulf Cooperation Council; MDT, multidisciplinary team; TNBC, Triple-negative breast cancer; VUS, variants of unknown significance

### 5.3 Treatment

Treatment of *BRCA* mutated early-stage BC is complex and involves the combination of local modalities, and systemic anticancer treatments delivered in diverse sequences.

#### 5.3.1 Surgery

Breast-conserving surgery or mastectomy are the primary treatment options for patients with BRCA mutated early BC (77), similar to sporadic BC (53). The choice of surgery depends on factors such as age, tumor size, location, TNM stage, and patient preferences (77). BCS is the optimal surgical choice when the tumor is small and is localized to one part of the breast (53). Mastectomy is indicated for the patients who choose to undergo this procedure over BCS or where there is an inability to achieve negative surgical margins after multiple resections or received prior radiation to the chest wall/breast or other contraindications to radiotherapy (RT) (41). Retrospective studies that evaluated long-term outcomes in *BRCA1/2* carriers have found no significant difference in RFS, breast cancer-specific survival (BCSS), or OS, between BCS and mastectomy; however, an increased risk of local recurrence was reported for BCS (78–81). Similar findings were reported in a systematic review by Co et al. (77), that compared survival outcomes and recurrence rates between *BRCA* mutation carriers who received BCS and those who received mastectomy. Overview of studies that evaluated BCS and mastectomy in early-stage BC patients with *BRCA* mutations are presented in **Supplementary Table S2** (77–81). Based on the available evidence, the researchers have indicated that BCS could be a good choice for *BRCA* mutation carriers, as long as they receive appropriate counseling and have rigorous follow-up (77).

Gentile et al, suggested that young *BRCA*-mutated patients with small tumors may not need an up-front mastectomy (82). Although data from the Danish Breast Cancer Group, reported a reduced risk of death for risk-reducing contralateral mastectomy (RRCM) (adjusted OS hazard ratio: 0.42, p=0.01) (83). A systematic review and meta-analysis by Fayanju et al. reported that RRCM may not necessarily result in improvement of OS, despite reducing the risk of contralateral BC (84). Limited data are available on the survival impact of RRCM in *BRCA* mutated patients with unilateral BC. For patients who require a mastectomy, breast reconstruction (immediate or delayed) could be an option (53). The nipple-sparing mastectomy has proven to be safe in patients carrying *BRCA* mutations, as both a therapeutic option and in terms of risk-reduction, due to its minimal local recurrence rates compared to a modified radical mastectomy (53, 85), although more data and longer follow-up are needed.

#### 5.3.2 Radiotherapy

A meta-analysis on randomized controlled clinical trials (RCTs) demonstrated a significant reduction in the 10-year risk of recurrence (absolute reduction 15.7%, 95% CI 13.7-17.7, 2p<0.00001) and 15-year risk of BC death (absolute reduction 3.8%, 1.6-6.0, 2p=0.00005) in patients with early BC who received whole breast irradiation after BCS compared with those who received BCS alone (86). Researchers reported that tumors harboring *BRCA* mutations could be sensitive to RT, because ionizing radiation has the ability to induce double standard breaks (DNBs) in DNA, and *BRCA* genes play a key role in repairing such DNBs (68). Multiple studies have reported that the risk of local recurrence after BCS and RT is comparable between patients with *BRCA*-mutated BC and those with sporadic BC (22, 87, 88). Similarly, studies have proven the equivalence in the survival of patients with *BRCA* mutations between BCS with RT vs mastectomy (78) or BCS vs mastectomy with RT (81). No evidence of impaired survival and toxicity related to irradiation has been observed in patients with *BRCA* mutations, suggesting that RT may be safe in these patients and should not be withheld (89, 90). Pierce et al (78) compared 10-year rates of IBTR/events after BCS and RT among *BRCA1/2* mutation carriers and women with sporadic BC and found no statistically significant difference. An overview of studies that evaluated adjuvant RT efficacy in BC patients with *BRCA* mutations is presented in **Supplementary Table S3** (78, 81, 87–89).

#### 5.3.3 Chemotherapy

Chemotherapy is recommended in the vast majority of TNBC, HER2-positive BC, and in high-risk luminal-like HER2-negative BC (53). The most frequently used regimen includes taxanes and/or anthracyclines but in selected patients, cyclophosphamide/5-fluorouracil (5-FU)/methotrexate may still be used (53). The taxanes, namely paclitaxel and docetaxel, play a significant role in the therapeutic management of BC. Patients with hormone receptor (HR) negative BC carrying *BRCA1* mutations exhibited less sensitivity to taxane chemotherapy than non-*BRCA1* mutation carriers with HR negative BC (91). Conversely, in patients with HR-positive BC with *BRCA1* and *BRCA2* mutations and sporadic cases, similar sensitivities were reported with taxane therapy (91). In the Arun et al. study, *BRCA1* mutation carriers showed higher pathological complete response (pCR) (46% vs 22%) compared to patients with sporadic BC, when treated with the combination of anthracycline-taxane, in neoadjuvant settings (92). Multiple studies have indicated that *BRCA1/2* mutation carriers are more prone to exhibiting a favorable response to neoadjuvant anthracycline-based regimens (92–94) or single-agent cisplatin (95), as evidenced by a higher rate of achieving pCR. In the INFORM trial, neoadjuvant single-agent cisplatin did not yield superior pCR rates when compared to the combination of doxorubicin and cyclophosphamide in patients with HER2 negative early BC who carry *BRCA1/2* mutations (96). In the TNT trial carboplatin demonstrated a markedly greater response in patients with *BRCA* mutated and TNBC as compared to docetaxel (20).

Studies that explored the utilization of platinum agents in neoadjuvant settings in patients with BRCA-mutated early-stage TNBC demonstrated high pCR rates with the addition of platinum agents to standard chemotherapy regimens (anthracycline, cyclophosphamide, taxanes) (97–99). Zang et al., reported improved recurrence‐free survival (RFS) and OS rates among *BRCA1/2*-mutated TNBC patients when carboplatin is added to standard anthracycline-taxane-based neoadjuvant chemotherapy (NACT) (100). On the contrary, in the GeparSixto trial and Brightness trial no additional benefit was observed in patients with BRCA-mutated TNBC with the addition of a platinum agent (carboplatin) to NACT (99, 101). An overview of studies that evaluated chemotherapy efficacy in BC patients with *BRCA* mutations are presented in **Supplementary Table S4** (91, 92, 94, 99–101).

#### 5.3.4 Endocrine therapy

Endocrine therapy (ET) is a common treatment option for early-stage BC. It is often used as adjuvant therapy after surgery to reduce the risk of BC recurrence, or as neoadjuvant therapy to shrink the tumor before surgery (53). Adjuvant tamoxifen (given for 5 years) showed a 31% decrease in mortality rate from BC in patients with estrogen receptor (ER)-positive BC and proved to be superior to 1 or 2 years of tamoxifen treatment (102). However, in patients with a *BRCA*-mutated HR-positive BC, ET exhibited a lower survival rate in comparison to their counterparts who do not possess the *BRCA* mutation (103). Empirical evidence indicates that tamoxifen use is associated with a reduction in contralateral BC risk among *BRCA* mutation carriers (104, 105), with a suggested influence on both ER-positive and -negative disease.

#### 5.3.5 Cyclin-dependent kinase 4/6 inhibitor

In MonarchE phase III trial, abemaciclib (cyclin-dependent kinase 4 and 6 inhibitor plus ET demonstrated superior invasive DFS (iDFS) compared with ET alone (hazard ratio: 0.75; 95% CI, 0.60 to 0.93, p = 0.01), with 2-year iDFS rates of 92.2% versus 88.7%, respectively in patients with HR-positive, HER2 negative, high-risk (≥ 4 positive nodes; 1–3 nodes involved and at least one of the following: tumor size ≥5 cm, histologic grade 3, or central Ki-67 ≥20%) early BC, in adjuvant settings (106). However, information on the *BRCA*m status of the patients who participated in the MonarchE trial is not available. The international guidelines endorse abemaciclib for HR-positive, HER2-negative germline *BRCA*m carriers who have undergone surgery first and have 1–3 positive nodes (107).

#### 5.3.6 Poly(ADP‐ribose) polymerase inhibitors

With the promising results for PARP inhibitors (Olaparib, talazoparib) in the treatment of *BRCA*-mutated BC in metastatic/advanced settings (108, 109), clinical trials are investigating their potential role in early-stage disease, as monotherapy or with other cytotoxic agents or with immunotherapy in neoadjuvant and adjuvant settings. Currently, the PARP inhibitor, olaparib is approved for the adjuvant treatment of adult patients with germline *BRCA*-mutated HER2-negative high-risk early BC who have been treated with neoadjuvant or adjuvant chemotherapy based on the results of the OlympiA trial (110, 111).

OlympiA was a double-blinded, phase III trial conducted to evaluate the safety and efficacy of adjuvant olaparib therapy versus placebo in high-risk, germline *BRCA*-mutated, HER2-negative early BC who received local treatment and neoadjuvant or adjuvant chemotherapy (at least 6 cycles anthracyclines or/and taxanes. OlympiA examined four patient populations considered to have HER2-negative disease at high risk of recurrence, and inclusion criteria varied based on tumor subtype and therapy setting (**Table 2**) (111).

**Table 2:** High-risk patient populations in the OlympiA Trial (111).

HER2 negative disease	Prior therapy	High-risk criteria
TNBC	Neoadjuvant	Non-pCR
	Adjuvant	≥pT2 or ≥pN1
HER2 negative HR-positive disease	Neoadjuvant	Non-pCR and CPS + EG score ≥3
	Adjuvant	≥4 LN+

CPS + EG, Combined Positive Score; clinical-pathologic staging system incorporating estrogen receptor-negative disease and nuclear grade 3 tumor pathology HR, Hormone receptor; HER2, Human epidermal growth factor receptor 2; LN, Lymph node; pCR, Pathological complete response; TNBC, Triple negative breast cancer.

At the initial interim analysis (111), a significant decrease in the disease recurrence or death was observed with the use of olaparib, successfully achieving the primary objective of the study (hazard ratio 0.58; p<0.001). In the second-interim analysis, a statistically significant and clinically meaningful improvement in OS was observed with olaparib compared with placebo (hazard ratio: 0.68; p=0.009) with an absolute improvement in 4-year OS of 3.4% (89.8% olaparib; 86.4% placebo) (112). The survival benefit of olaparib was observed irrespective of germline *BRCA* status, HR status, prior platinum use, and adjuvant chemotherapy or NACT (112). The international guidelines have updated their BC treatment guidelines to include treatment with the PARP inhibitor olaparib for one year after completing chemotherapy, surgery, and radiation (if used) to improve outcomes in patients with an inherited mutation in *BRCA1/2* with early-stage, HER2-negative BC who have a high risk for recurrence (113).

In the phase II study, talazoparib monotherapy elicited pCR rates that were comparable to those observed with combination anthracycline and taxane-based chemotherapy regimens when used in the neoadjuvant settings in patients with *BRCA1/2* positive, early HER2-negative BC (114). In the phase II single-arm NEOTALA trial, talazoparib yielded promising pCR rates in patients with *BRCA* mutated early BC comparable to those historically observed with combination anthracycline- and taxane-based chemotherapy regimens (115).

In the phase II ISPY-2 trial, veliparib plus carboplatin and paclitaxel showed better pathological complete response (51%, 95% CI, 36% to 66%) in early-stage TNBC, compared with paclitaxel alone (26%, 95% CI, 9% to 43%) (116). Similar pCR rates were demonstrated with the addition of veliparib plus carboplatin or carboplatin alone to NACT in the BrighTNess trial (53% vs 31%) (99). After a median follow-up of 4.5 years, event-free survival (EFS) was significantly improved for the veliparib, carboplatin, plus paclitaxel group relative to the paclitaxel alone group (hazard ratio: 0.63, p=0.02), but no difference was observed between veliparib, carboplatin, plus paclitaxel group and the carboplatin plus paclitaxel group (hazard ratio: 1.12, p= 0.62) (117). The addition of veliparib did not impact the EFS (117). Clinical trial data on oral PARP inhibitors is presented in **Table 3** (111, 112, 114–117).

**Table 3:** Clinical trials of oral PARP inhibitors for early-stage breast cancer in the neoadjuvant/adjuvant settings.

Author and year	Patient population (N)	Study design	Treatment modality	Key outcomes
OlympiA trial (111, 112)	High-risk, gBRCAm, HER2 negative breast cancer N= 1836	Phase III, Randomized, double-blind trial	1 year of oral olaparib or placebo	Olaparib (n=911) vs Placebo (904) At a prespecified event-driven interim analysis (median follow-up of 2.5 years): ▪ 3-year iDFS: 85.9% vs 77.1% ▪ Invasive disease or death: HR 0.58; 99.5% CI, 0.41 to 0.82; p<0.001 ▪ Deaths (n): 59 vs 86; HR: 0.68; 99% CI, 0.44 to 1.05; p=0.02 At secondary interim analysis at median follow up of 3.5 years: ▪ OS: HR: 0.68; 98.5% CI, 0.47 to 0.97; p= 0.009; 4-year OS rate: 89.8% vs 86.4% ▪ 4-yr iDFS: 82.7% vs 75.4% ▪ 4-year DDFS: 86.5% vs 79.1% ▪ SAEs: 8.7% vs 8.6 ▪ AE’s led to permanent discontinuation: 10.8% vs 4.6 ▪ Common reasons for olaparib discontinuation: Nausea (2.2%), anemia (1.8%), fatigue (1.6%), and decreased neutrophil count (1.0%)
I-SPY 2 (116)	TNBC, stage II or III N=72	Phase II	Veliparib- Carboplatin (AUC6, q3 weeks) and standard NACT vs. standard NACT alone	Veliparib (n=72) vs standard NACT alone (n=44) ▪ pCR = 51% vs. 26%
BrighTNess (117)	Stage II–III TNBC N= 634	Phase III Randomized, double-blind, placebo-controlled trial	Weekly paclitaxel 12 doses +/- Carboplatin AUC6 (q3 weeks, 4 cycles) +/- veliparib	Carboplatin plus veliparib with paclitaxel (n=316) vs carboplatin with paclitaxel (n=160) vs paclitaxel (n=158) At 4.5 yrs follow up: Carboplatin + veliparib + paclitaxel versus paclitaxel alone ▪ EFS: HR: 0.63, 95% CI, 0.43 to 0.92, p= 0.02 ▪ OS: HR: 0.82, 95% CI 0.48 to1.38, p= 0.45 Carboplatin + veliparib + paclitaxel vs carboplatin + paclitaxel ▪ EFS: HR: 1.12, 95% CI 0.72 to 1.72, p=0.62 ▪ OS: HR: 1.25, 95% CI 0.70 to 2.24, p= 0.46
NCT03499353 (114)	HER2 negative, gBRCAm stage I to III N = 20	Pilot study	Talazoparib for 6 months	▪ pCR = 53% ▪ RCB 0–I = 63% ▪ grade 3 AEs (n): anemia (8), neutropenia (3) ▪ grade 4 AEs (n=1): Thrombocytopenia (1)
NEOTALA trial NCT03499353 (115)	gBRCAm early-stage TNBC	Phase II, single-arm, open-label study	Talazoparib	Evaluable population and ITT population ▪ pCR rate: 45.8% and 49.2% respectively ▪ RCB 0/I rate: 45.8% and 50.8% respectively

AEs, Adverse events; AUC, Area under the curve; EFS, Event free survival; CI, Confidence interval; gBRCAm, Germline BReast CAncer gene mutation; iDFS, Invasive disease survival; ITT, Intention-to-treat; OS, Overall survival; HR, Hazard ratio; HER2, Human epidermal growth factor receptor 2; NACT, Neoadjuvant chemotherapy; RCB, Residual cancer burden; pCR, Pathological complete response; TNBC, Triple negative breast cancer; SAEs, Serious adverse events; q3week, Every-3-week.

#### 5.3.7 Combination of PARP inhibitors and immunotherapy agents

Multiple clinical trials have assessed the efficacy of combining PARP inhibitors and immune checkpoint inhibitors (ICIs) in the context of metastatic cancer. These trials include MEDIOLA (118), TOPACIO (119), KEYLYNK-007 (120), and JAVELIN PARP Medley (121). The results of these trials have demonstrated a favorable toxicity profile for pembrolizumab (119, 120), durvalumab (118), and avelumab (121). The combination of PARP inhibitors and ICIs may emerge as a promising therapeutic strategy for patients harboring *BRCA* mutations. These tumors appear as more immunogenic due to their higher levels of tumor-infiltrating lymphocytes, higher mutational burden, and expression of immune checkpoint inhibitory molecules compared to BCs without *BRCA* mutations. Encouraged by results for ICIs from the metastatic setting, ICIs are now being assessed for the treatment of advanced mutated BC and early-stage BC with *BRCA* mutations (DORA trial, DOLAF, KEYLYNK trial, NCT03329937, NCT03150576, and NCT03499353).

In the phase II ISPY-2 trial, neoadjuvant durvalumab and olaparib exhibited superior efficacy in terms of pCR over standard NACT in HER2-negative BC, particularly in a highly sensitive subset of high-risk HR-positive, HER2-negative patients (64% vs 22%) (122). Although it remains uncertain whether the incorporation of immunotherapy alongside PARP inhibitors yields superior outcomes in comparison to single-agent PARP inhibitors, safety data obtained in the metastatic context indicate that the combination is well-tolerated. Despite the lack of empirical evidence, data extrapolation consequently advocates for the co-administration of adjuvant pembrolizumab and olaparib in high-risk patients with residual disease following chemo-immunotherapy.

## 6 Healthcare infrastructure in the GCC region for managing *BRCA*-mutated early-stage BC

In recent years, GCC countries have made significant progress in enhancing the healthcare infrastructure for BC management. Most of these countries have specialized cancer centers (King Faisal Specialist Hospital & Research Centre in KSA, Tawam Hospital Comprehensive Cancer Center in UAE, National Oncology Centre at the Royal Hospital in Oman, Kuwait Cancer Control Center in Kuwait, The Bahrain Oncology Center at King Hammad University Hospital in Bahrain) equipped with advanced technology and staffed by MDT healthcare professionals (123–128). These centers function as central points for cancer diagnosis, treatment, and survivorship care, providing patients with comprehensive and specialized services. With the exception to Oman, the healthcare infrastructure in these countries is equipped with state-of-the-art diagnostic tools like mammography, ultrasound, MRI, PET and molecular testing (123–128). In Saudi Arabia, oncology services are offered through various public institutions (126). In UAE, several general oncology care services have been initiated across the nation to enable cancer patients to access healthcare facilities closer to their homes (125). In Kuwait and Bahrain, all the general hospitals are equipped with a radiology department along with molecular imaging and nuclear medicine (123, 124). In UAE, radiotherapy facilities are located across the country and offer advanced treatment options (125).

Besides in countries like KSA, UAE and Qatar, cancer care is provided free of charge to all their citizens through health insurance and non-profit organizations to non-citizens (125, 126, 128). Such care includes laboratory tests, clinical imaging, systemic anti-cancer therapies, surgery, and radiotherapy. Bahraini citizens, on the other hand, receive free treatment at public hospitals. Most GCC nations provide an extensive array of treatment choices such as surgery, chemotherapy, radiation therapy, hormone therapy and targeted therapy guaranteeing individualized and efficient healthcare for patients (123–127).

## 7 Challenges in management of *BRCA*-mutated early-stage BC

Limited availability of specialized BC centers and oncology services in certain GCC countries can impede timely access to comprehensive care, including diagnosis, treatment, and support services. Within GCC countries, geographic disparities in healthcare infrastructure and resources may result in unequal access to BC care, especially for patients residing in remote or rural areas far from major healthcare facilities. In most GCC countries patients have access to all historically used drugs; however, there is a wide disparity in accessing novel targeted therapies such as abemaciclib olaparib, and talazoparib due to lack of approval or non-reimbursement, or high costs. For example, in UAE and Saudi Arabia, drugs like abemaciclib, olaparib are approved for *BRCA* mutated BC (7). However, in countries like Kuwait they are not accessible yet for management of early-stage BC. Hence, governments may need to take efforts to ensure that novel drugs are readily available to patients from these countries.

Financial barriers, such as high treatment costs, insurance limitations, and out-of-pocket expenses, may present challenges for patients, particularly non-citizens and those without sufficient insurance coverage, despite efforts to provide free or subsidized cancer care for citizens. Besides, patient factors such as adherence to treatment protocols, including medication regimens and follow-up appointments, can be difficult due to factors such as treatment-related side effects, logistical challenges, and cultural beliefs about illness and treatment. Cultural beliefs and social stigma surrounding cancer, specifically BC, can influence patient decision-making, treatment-seeking behavior, and disclosure of diagnosis. Fear, misconceptions, and cultural taboos may contribute to delayed presentation, reluctance to seek medical help, and adherence issues. Gender norms and cultural expectations regarding women’s roles and health-seeking behavior can impact access to BC care. Cultural factors related to modesty, privacy concerns, and family dynamics may affect women’s ability to access screening services, discuss symptoms, and seek timely medical care.

Thus, it is imperative to increase awareness among people toward BC, genetic testing, and advocacy for insurance coverage. Additionally providing support services to promote treatment adherence and address psychosocial needs is essential.

## 8 Expert panel recommendations for the management of *BRCA* mutated early BC in germline *BRCA* carriers

**Figure 1:**
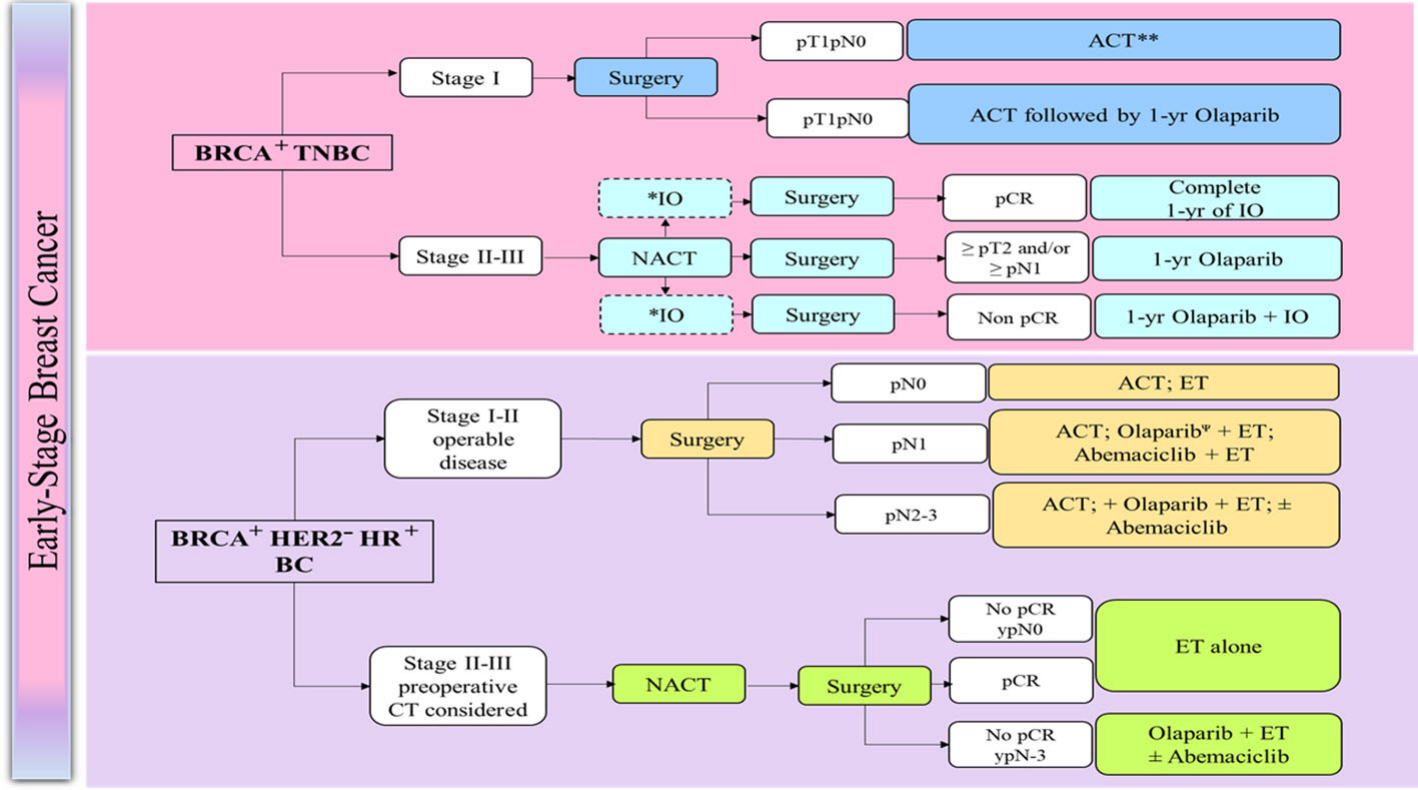
Management of early breast cancer in germline *BRCA1/2* mutation carriers (129). ACT, Adjuvant chemotherapy; BRCA, BReast CAncer gene; CT, chemotherapy; CPS + EG, Combined Positive Score; clinical-pathologic staging system incorporating estrogen receptor-negative disease and nuclear grade 3 tumor pathology; ER, estrogen receptor; ET, endocrine therapy; HR, Harmone receptor; HER2, human epidermal growth factor receptor 2; NACT, Neoadjuvant chemotherapy; N0, node-negative; LN, Lymph node; pCR, Pathological complete response; TNBC, triple-negative breast cancer. *: PDL1 positive; **: case-by case for pT1a, Ψ: Preferred after chemotherapy, case-by-case for pT1a.

Expert panel recommendations for the management of early BC are presented in **Box 2** and the treatment algorithms are presented in **Figure 1** (129).

Box 2 **Panel recommendations for the management of early BC in Germline *BRCA* carriers.**

  ▪ BCS is the preferred local treatment option for the majority of *BRCA*-mutated early BC patients, with the use of oncoplastic techniques, to maintain good cosmetic outcomes in technically challenging cases, when needed.

▪ After surgical resection, careful assessment of resection margins is essential. No tumor at the inked margin is recommended for either *in situ* disease or invasive BCs and >2 mm for *in situ* disease is recommended (41).

▪ Prophylactic contralateral mastectomy should be considered for patients with a very early disease with T0, T1, or M0 with germline *BRCA* mutations.

▪ Breast reconstruction should be available and proposed to all women requiring mastectomy. Immediate breast reconstruction should be offered to the vast majority of patients, except for those presenting with inflammatory cancer.

▪ The optimal reconstruction technique for each patient should be discussed individually taking into account anatomic, treatment and patient-related factors and preferences.

▪ Postoperative RT is strongly recommended after BCS. Post-mastectomy RT is recommended for high-risk patients, including those with involved resection margins, involved axillary lymph nodes and T3–T4 tumors; it should also be considered in patients with 1–3 positive axillary lymph nodes.

▪ Patients fulfilling the OlympiA criteria are considered high-risk patients for recurrence.

▪ The panel recommends offering 1-year of adjuvant olaparib for patients with high-risk, early-stage HER2-negative BC with germline *BRCA* mutations after completion of neoadjuvant chemotherapy and local treatment, including radiation.

▪ In patients with TNBC with germline *BRCA* mutations and PDL1 expression who did not achieve pCR with neoadjuvant treatment, a combination of pembrolizumab with olaparib is recommended. However, there is no clinical evidence to support the recommendation.

BC, Breast cancer; BCS, Breast conservative surgery; BRCA, BReast CAncer gene; HER2, Human epidermal growth factor receptor 2; pCR, pathologic complete response; RT, Radiotherapy.

## 9 Conclusion

*BRCA* testing is an important and critical diagnostic tool in the early-stage BC care as it enables personalized treatment plans to improve patient outcomes. With continued advancements in research and technology, the role of *BRCA* testing in the management of BC is likely to expand further, offering new opportunities for early detection, risk reduction, and personalized care for patients with *BRCA*-mutated BC. With the availability of comprehensive and cost-effective genetic testing for germline *BRCA1/2* mutations and the valuable information it provides for treatment options, it may be reasonable to consider *BRCA* testing beyond previously established selection criteria. The inclusion of olaparib as an adjuvant therapy for early BC patients with *BRCA* mutations marks a significant advancement in the treatment of this patient population. It is imperative for surgical/medical oncologists to consider it in the locoregional systemic management of early BC for improved patient outcomes.

## Author contributions

HA-S: Conceptualization, Writing – original draft, Writing – review & editing. AA: Conceptualization, Writing – original draft, Writing – review & editing. FA: Conceptualization, Writing – original draft, Writing – review & editing. FC: Conceptualization, Writing – original draft, Writing – review & editing. ST: Conceptualization, Writing – original draft, Writing – review & editing. SHT: Conceptualization, Writing – original draft, Writing – review & editing. SK: Conceptualization, Writing – original draft, Writing – review & editing. SO: Conceptualization, Writing – original draft, Writing – review & editing. OA: Conceptualization, Writing – original draft, Writing – review & editing.
