# Supplementary figures and images for: BRCA testing and management of BRCA-mutated early-stage breast cancer: a comprehensive statement by expert group from GCC region
Source: Front Oncol. 2024 Apr 25;14:1358982. doi: 10.3389/fonc.2024.1358982 (PMC11080009; doi:10.3389/fonc.2024.1358982)

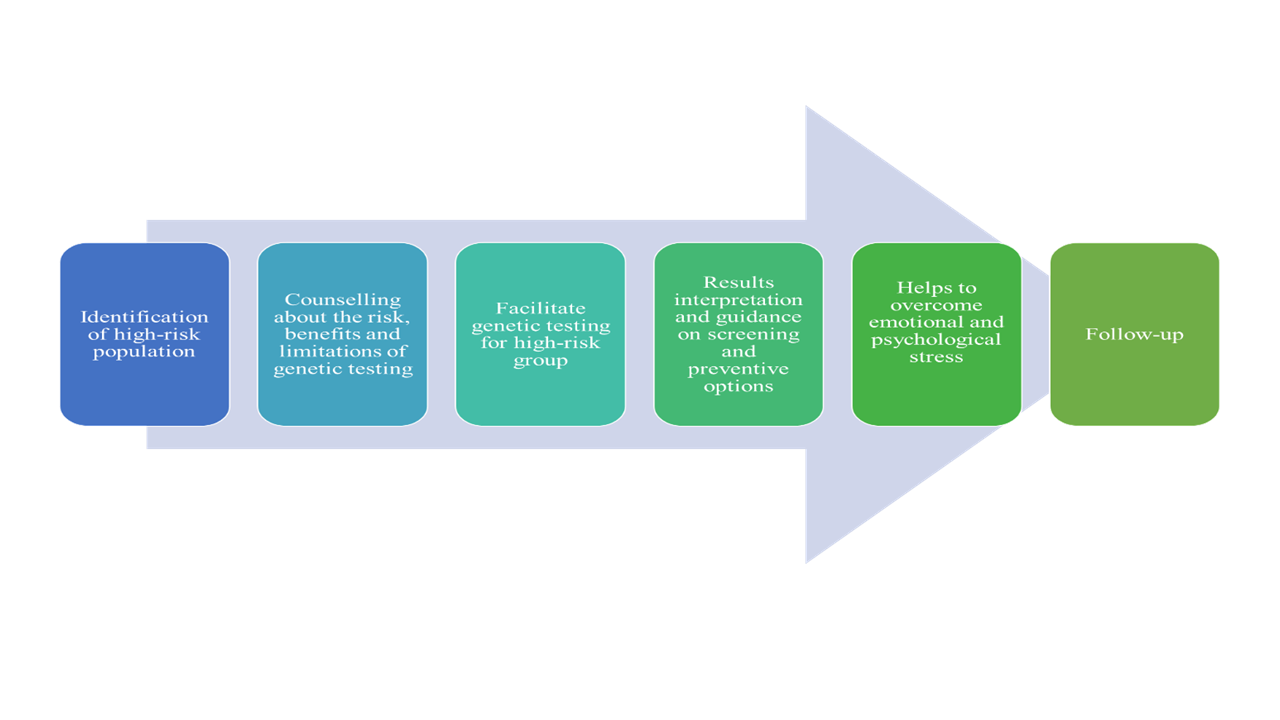

Supplement: Supplementary file 1 [file Image_1.tif]
