# Supplementary material for: BRCA testing and management of BRCA-mutated early-stage breast cancer: a comprehensive statement by expert group from GCC region
Source: Front Oncol. 2024 Apr 25;14:1358982. doi: 10.3389/fonc.2024.1358982 (PMC11080009; doi:10.3389/fonc.2024.1358982)
Supplement: Supplementary file 2 [file Table_1.docx]

# Supplementary data:

**Supplementary Figure Captions:**

**Supplementary Figure S1: Role of Genetic Counselor in Early BC Management**

**Supplementary Tables:**

**Supplementary Table S1. Age-standardized Incidence and Mortality Rates of BC in Women in GCC Region in 2020**

| **Countries** | **ASIR (per 100,000)** | | ***Cum risk** | **ASMR (per 100,000)** | | ***Cum risk** |
| --- | --- | --- | --- | --- | --- | --- |
|  | **Both sex** | **Females** |  | **Both sex** | **Females** |  |
| GCC region | 34.4 | 34.4 | 5.39 | 10.6 | 10.6 | 2.38 |
| Saudi Arabia | 28.8 | 28.8 | 4.38 | 8.9 | 8.9 | 1.96 |
| Bahrain | 44.2 | 44.2 | 7.03 | 13.6 | 13.6 | 3.27 |
| Kuwait | 50.3 | 50.3 | 9.66 | 17.0 | 17.0 | 4.95 |
| Oman | 38.5 | 38.5 | 5.16 | 14.5 | 14.5 | 2.50 |
| Qatar | 42.7 | 42.7 | 6.94 | 13.2 | 13.2 | 2.78 |
| UAE | 58.5 | 58.5 | 12.32 | 16.6 | 16.6 | 5.44 |

Source: Globocan data(1); *: Cumulative risk is applicable for both sexes as well as females; ASIR, Age-standardized incidence rate; ASMR, Age-standardized mortality rates; BC, Breast cancer; Cum, Cumulative; GCC, Gulf Cooperation Council; UAE, United Arab Emirates

**Supplementary Table S2**: **Overview of Studies that Evaluated BCS and Mastectomy in Early-stage BC Patients with BRCA Mutations**

| **Author and year** | **Patient population** | **Study design and number of patients** | **Key outcomes** |
| --- | --- | --- | --- |
| Pierce et al., 2010(78) | Stage I-III, invasive BC patients carrying BRCA mutations | Retrospective study  N= 655 (BCS=302; mastectomy=353) | BCS vs Mastectomy   - OS rates at 10 yrs and 15 yrs: 92.1% and 87.3% vs 91.8% and 89.8% (p=0.73) - BCSS at 10 yrs and 15 yrs: 93.6% and 91.7% vs 93.5% and 92.8% (p=0.85) - Estimated cumulative risk at 10 yrs and 15 yrs for local failure: 10.5 and 23.5% vs. 3.5% and 5.5% (p<0.0001). |
| Nilsson et al., 2014(79) | Stage I-III, invasive BC patients carrying BRCA1 or BRCA2 mutations | Retrospective study  N=162 (BCS=45; Mastectomy=118) | BCS vs Mastectomy   - Estimated cumulative risk of local recurrence at 15 yrs: 32% vs 9% - Breast cancer deaths at 15 yrs: 34% vs 29% |
| Van den Broek., 2019(80) | Invasive BC patients with BRCAm | Retrospective study  N = 5820, (BRCA1m carriers = 191; BRCA2m carriers = 70) | BCS vs Mastectomy  In BRCA1m carriers:   - OS: HR= 0.80, 95% CI, 0.42 to 1.51, p = 0.50 - BCSS: HR= 0.75, 95% CI, 0.34 to 1.65, p= 0.47 - Numbers for BRCA2m were insufficient to draw conclusions.   BRCA1m carriers vs noncarriers:   - The rate of local recurrences after BCS (10-year risk) 7.3% vs 7.9% |
| Wan et al., 2021(81) | Invasive breast cancer (stages I-III), which also includes 491 BRCA1/2 mutation carriers | Retrospective cohort study  Overall population: (N=8396) among them 491 are BRCA1/2 mutation carriers | BCS vs Mastectomy (at median follow up of 7.5yrs):  In BRCA1m carriers:   - BCSS: HR=1.12, 95% CI, 0.30 to 4.22; p = 0.87 - OS: HR=1.29, 95% CI, 0.39 to 4.24; p = 0.68 - RFS: HR:1.04, 95% CI, 0.46 to 2.35; p=0.93 - IBTR rates: 1.4% vs 3.6% - Rates of contralateral BC: 13.7% vs 11.9%   In BRCA2m carriers:   - BCSS: HR: 0.90, 95% CI, 0.23 to 3.60; p = 0.89 - OS: HR: 0.60, 95% CI, 0.16 to 2.20; p =0.44 - RFS: HR: 0.73, 95% CI, 0.36 to 1.48; p=0.38 - IBTR rates: 7.5% vs 3.8% - Rates of contralateral BC: 6.6% vs 19.8% |

BC, Breast cancer; BCS, breast-conserving surgery; BCSS, breast cancer–specific survival; BRCAm, BReast CAncer gene mutation; BRCA1m, BReast CAncer gene 1 mutation; BRCA2m, BReast CAncer gene 2 mutation; CI: Confidence interval; DRFS, distant recurrence–free survival; HR, hazard ratio; OS, overall survival; RFS, recurrence-free survival; RT, radiotherapy. IBTR, Ipsilateral breast tumor recurrence

**Supplementary Table S3: Overview of Studies that Evaluated Adjuvant RT in** **BC Patients with BRCA Mutations**

| **Author and year** | **Patient population** | **Study design and number of patients** | **Key outcomes** |
| --- | --- | --- | --- |
| Shanley et al., 2006(89) | BRCA1/2 mutation carriers with breast cancer and sporadic BC | Retrospective case control study BRCA1/2=55 and control=55 | Medians follow up of 6.75 yrs for BRCA carriers and 7.75 yrs for controls   - Increased recall of acute pain in BRCA carriers than control (% any pain difference: 21.1%; 95% CI, 3.7 to 38.7) |
| Pierce et al., 2006(87) | BRCA1/2 mutation carriers with breast cancer and controls with sporadic breast cancer | Retrospective case control study BRCA1/2=160 and control=445 | Median follow up of 7.9 yrs  BRCA carriers vs control   - Overall rates of IBTR at 10 yrs and 15 yrs: 12% (95% CI, 9 to 15) and 24% (95% CI, 17 to 33) vs 9% (95% CI, 7 to 10) and 17% (95% CI, 12 to 21) - Reduction in IBTR after oophorectomy: HR=0.55; p = 0.44 - In patients with local recurrence, the median time to IBTR: 8.7 yrs vs 4.7 yrs; p = 0.01 - Recurrent lesions located in quadrants: 60% vs 29%; p = 0.04 - Recurrence at any site: 0% vs 29% |
| Brekelmans et al., 2006(88) | Invasive breast cancer with BRCA1m and sporadic mutation | Retrospective, case-control study  BRCA1m carriers=223 and controls=446 | Median follow up of 5.1 yrs  BRCA1-associated and sporadic tumors   - ipsilateral BC recurrence: HR= 0.7; p = 0.24 - DDFS: HR= 1.2; p = 0.37 - BCSS: HR= 1.3; p = 0.23 |
| Pierce et al., 2010(78) | Stage I-III, invasive BC patients carrying BRCA mutations | Retrospective study  N= 655 (BCS=302; mastectomy=353) | BCS+RT vs Mastectomy   - OS rates at 10 yrs and 15 yrs: 92.1% and 87.3% vs 91.8% and 89.8%; p=0.73) - BCSS at 10 yrs and 15 yrs: 93.6% and 91.7% vs 93.5% and 92.8%; p=0.85)   Estimated cumulative risk at 15 yrs for local failure: 23.5% vs 5.5%; p<0.0001 |
| Wan et al., 2021(81) | Invasive breast cancer (stages I-III), which also includes 491 BRCA1/2 mutation carriers | Retrospective cohort study  Overall population: (N=8396) among them 491 are BRCA1/2 mutation carriers | Median follow up of 7.5 yrs  BCT vs Mastectomy +RT  In BRCA1m carriers:   - BCSS: HR=1.31, 95% CI, 0.12 to 14.49; p = 0.83 - OS: HR=1.31; 95% CI, 0.13 to 13.33; p = 0.82 - RFS: HR=0.64; 95% CI, 0.17 to 2.44; p = 0.52 - DRFS: HR=0.63; 95% CI 0.15 to 2.63; p = 0.53 - IBTR rates: 1.4% vs 0% - Rates of contralateral BC: 13.7% vs 20.0%   In BRCA2m carriers:   - BCSS: HR=0.76, 95% CI, 0.13 to 4.50; p = 0.77 - OS: HR=0.98; 95% CI, 0.18 to 5.21; p = 0.98 - RFS: HR=0.66; 95% CI, 0.23 to 1.90; p = 0.44 - DRFS: HR=0.58; 95% CI 0.18 to 1.86; p = 0.36 - IBTR rates: 3.8% vs 1.5% - Rates of contralateral BC: 6.6% vs 11.9% |

BC, Breast cancer; BCS, breast-conserving surgery; BCSS, breast cancer–specific survival; BRCA, germline BReast CAncer gene; BRCAm, germline BReast CAncer gene mutation; BRCA1m, BReast CAncer gene 1 mutation; BRCA2m, BReast CAncer gene 2 mutation; CI: Confidence interval; DDFS, Distant disease-free survival; DRFS, distant recurrence–free survival; HR, hazard ratio; IBTR, Ipsilateral breast tumor recurrence; OS, overall survival; RFS, recurrence-free survival; RT: Radiotherapy; vs, Versus; yrs, years

**Supplementary Table S4: Overview of Studies that Evaluated Chemotherapy in BC Patients with BRCA Mutations**

| **Author and year** | **Patient population** | **Study design and number of patients** | **Key outcomes** |
| --- | --- | --- | --- |
| Arun et al., 2011(92) | Breast cancer with or without BRCA1 or BRCA 2 mutations | Retrospective study  N=317 (Anthracycline-taxane–containing regimens with a taxane [n = 261], anthracycline-based regimens without a taxane [n = 40], or single-agent taxane [n = 16]) | Independent significant predictors for a pCR   - BRCA1 status: OR=3.16; 95% CI, 1.55 to 6.42; p = 0.002, - ER negativity: OR=1.96; 95% CI, 1.05 to 3.65; p = 0.03 - Concurrent trastuzumab use: OR=4.18; 95% CI, 2.04 to 8.57; p < 0.0001   5-yrs RFS rate (Median follow-up of 3.2 yrs)   - Overall: 74% (95% CI, 68 to 81) - Noncarrier: 73% (95% CI, 67 to 81) - BRCA1: 72% (95% CI, 59 to 88) - BRCA2: 93% (95% CI, 80 to 100)   BRCA1 carriers who achieved a pCR had better 5-year RFS (95% vs 53% p = 0.001) and OS (100% vs 75%; p = 0.01) rates than those who did not |
| Kriege et al., 2012(91) | Invasive breast cancer with BRCA1 or BRCA 2 and sporadic mutation | Retrospective study  BRCA1/2=48 and control=95  Docetaxel: every 3 weeks (100 mg/m^2^) or weekly (30-36 mg/m^2^),  Paclitaxel: every 3 weeks (175 mg/m^2^) or in a weekly regimen (90 mg/m^2^ on days 1, 8, and 15, repeated every 4 weeks) | BRCA1 associated patients vs sporadic mutations   - ORR: 23% vs 38% - PD: 60% vs 19%; p < 0.001 - Median PFS: 2.2 vs 4.9 months; p = 0.04   Hormone receptor-negative patients, BRCA1-associated patients vs sporadic mutations   - ORR: 20% vs 42% - PD: 70% vs 26%; p = 0.03 - Median PFS: 1.8 vs 3.8 months; p = 0.004   Hormone receptor-positive patients, BRCA1-associated patients vs sporadic mutations   - ORR: 36% vs 38% - PD: 28% vs 20% - Median PFS: 5.7 vs 5.7 months   Hormone receptor-positive patients, BRCA2-associated patients vs sporadic mutations   - ORR: 89% vs 38%; p = 0.02 - Median PFS: 7.1 vs 5.7 months |
| Wang et al., 2015(94) | TNBC | Retrospective study  N=956  Neoadjuvant anthracycline-based regimens | BRCA carriers vs non carriers   - pCR: 53.8% vs 29.7%, p < 0.001 - pCR: 57.1% vs 29.0%; p < 0.001 in patients treated with anthracycline or without taxane regimens - pCR: 40.0% vs 32.9%; p = 0.73 in patients treated with taxane regimens - Recurrence-free survival: adj HR, 0.92; 95% CI, 0.45–1.90; p = 0.82 |
| Hahnen et al., 2017(101) | TNBC | Randomized clinical trial, N=291  Neoadjuvant carboplatin therapy | BRCAm carriers vs non carriers   - - pCR rate in non‑carboplatin arm: 66.7% vs 36.4% (OR: 3.50; 95% CI, 1.39 to 8.84; p = 0.008)   Carboplatin therapy vs non‑carboplatin therapy   - pCR rate in BRCAm carriers: 65.4% vs 66.7%; OR, 0.94; 95% CI, 0.29 to 3.05; p=0.92 - DFS rates in BRCAm carriers: 73.5% (95% CI, 64.1 to 80.8) vs 85.3% (95% CI, 77.0 to 90.8); HR, 0.53; 95% CI, 0.29 to 0.96; p = 0.04 |
| Zhang et al., 2021(100) | BRCA1/2-mutated breast TNBC | Retrospective study  N= 1585  Anthracycline-taxane-based or anthracycline-taxane/carboplatin-based neoadjuvant chemotherapy | Median follow-up of 81 months  Anthracycline-taxane-based vs anthracycline-taxane/carboplatin-based therapy in BRCA1/2 mutation carriers   - 5-year RFS: 82.6% vs 47.9%; p = 0.024 - 5-year DRFS: 88.5% vs 46.9%; p = 0.010 - 5-year OS: 88.2% vs 49.9%; p = 0.036   Anthracycline-taxane/carboplatin-based vs anthracycline-taxane-based therapy in BRCA1/2 mutation carriers   - RFS: adj HR, 0.24; 95% CI, 0.06 to 0.91, p = 0.035 - DRFS: adj HR, 0.17; 95% CI, 0.03 to 0.80; p = 0.025 - OS: adj HR, 0.29; 95% CI, 0.06 to 1.49; p = 0.14 |
| BrighTNess(99) | Stage II–III TNBC | Phase III Randomized, double-blind, placebo-controlled trial  N= 634 (Carboplatin plus veliparib with paclitaxel [n=316]; carboplatin with paclitaxel [n=160]; paclitaxel [n=158]) | Carboplatin plus paclitaxel and veliparib vs paclitaxel vs carboplatin with paclitaxel   - pCR rate: 53% vs 31% p<0·0001   Carboplatin with paclitaxel vs carboplatin plus paclitaxel:   - pCR rate: 53% vs 58%, p=0·36 |

adj HR, adjusted hazard ratio; BC, Breast cancer; BCS, breast-conserving surgery; BCSS, breast cancer–specific survival; BRCA, BReast CAncer gene; BRCAm, BReast CAncer gene mutation; CI: Confidence interval; DRFS, distant recurrence–free survival; ER, estrogen receptor; HR, hazard ratio; OR, Odds ratio; ORR, objective response rate; OS, overall survival; RFS, recurrence-free survival; TNBC, Triple-negative breast cancer; pCR, pathologic complete response; PD, progressive disease; vs, Versus; yrs, years
